# Chicago Medical College

**Published:** 1886-04

**Authors:** 


CHICAGO MEDICAL COLLEGE.—The twenty-seventh annual commencement exercises of the Chicago Medical College, Medical Department of the Northwestern University, were held on Tuesday, March 23.

Rev. Joseph Cummings, D.D., LL.D., President of the University, conferred the degree of Doctor of Medicine upon thirty-six members ot the senior class. The *ad eundem* degree of Doctor of Medicine was conferred upon William Albert Dickey, M.D., Ezra Augustine Palmer, M.D. George E. Vance received the honorary degree of Doctor of Medicine.

Normal histology is studied in the microscopical laboratory during the first year.

During the past year a laboratory devoted to pathological histology has been created.

A determined effort is being made by the friends of the institution to establish a physiological laboratory.

During the past year instruction has been given in comparative embryology.

